# The Mathematical Modeling of the Host–Virus Interaction in Dengue Virus Infection: A Quantitative Study

**DOI:** 10.3390/v16020216

**Published:** 2024-01-31

**Authors:** Zhaobin Xu, Hongmei Zhang, Dongying Yang, Dongqing Wei, Jacques Demongeot, Qiangcheng Zeng

**Affiliations:** 1 School of Life Science, Dezhou University, Dezhou 253023, China; 2 School of Medicine, Dezhou University, Dezhou 253023, China; 3 School of Life Sciences and Biotechnology, Shanghai Jiao Tong University, Shanghai 200030, China; dqwei@sjtu.edu.cn; 4 Laboratory AGEIS EA 7407, Team Tools for e-Gnosis Medical, Faculty of Medicine, University Grenoble Alpes (UGA), 38700 La Tronche, France; jacques.demongeot@univ-grenoble-alpes.fr

**Keywords:** Dengue fever, antibody dynamics, antibody-dependent enhancement, viral load, mathematical modeling

## Abstract

Infectious diseases, such as Dengue fever, pose a significant public health threat. Developing a reliable mathematical model plays a crucial role in quantitatively elucidating the kinetic characteristics of antibody–virus interactions. By integrating previous models and incorporating the antibody dynamic theory, we have constructed a novel and robust model that can accurately simulate the dynamics of antibodies and viruses based on a comprehensive understanding of immunology principles. It explicitly formulates the viral clearance effect of antibodies, along with the positive feedback stimulation of virus–antibody complexes on antibody regeneration. In addition to providing quantitative insights into the dynamics of antibodies and viruses, the model exhibits a high degree of accuracy in capturing the kinetics of viruses and antibodies in Dengue fever patients. This model offers a valuable solution to modeling the differences between primary and secondary Dengue infections concerning IgM/IgG antibodies. Furthermore, it demonstrates that a faster removal rate of antibody–virus complexes might lead to a higher peak viral loading and worse clinical symptom. Moreover, it provides a reasonable explanation for the antibody-dependent enhancement of heterogeneous Dengue infections. Ultimately, this model serves as a foundation for constructing an optimal mathematical model to combat various infectious diseases in the future.

## 1. Introduction

Dengue fever is a viral disease transmitted by mosquitoes that affects a substantial proportion of the population residing in tropical and subtropical regions. The disease is caused by four closely related but distinct viruses, namely DENV-1, DENV-2, DENV-3, and DENV-4, and it is estimated that approximately 400 million cases of Dengue fever occur globally each year [1,2,3]. The severity of the disease is significantly influenced by the individual’s immunological status.

Unlike infections caused by other viruses such as SARS-CoV-2 and influenza, Dengue fever exhibits marked differences in IgM and IgG dynamics between primary and secondary infections. Clinical evidence suggests that during primary infection, IgM levels increase significantly, while IgG levels only undergo a slight increase. In contrast, in secondary infections, IgG levels experience a significant proliferation, with a higher peak level than IgM in most cases. Scientists have also utilized this feature to distinguish between primary and secondary infections [4,5,6]. Moreover, it is widely recognized that secondary infections confer more durable protection against homogenous reinfection.

Another intriguing phenomenon in Dengue virus infection is antibody-dependent enhancement (ADE), observed when an individual is reinfected with a heterogenous subtype. In addition to conferring lifelong protection against a specific serotype, IgG antibodies can cross-react with heterologous DENV serotypes [7,8,9,10,11]. Rather than neutralizing the new Dengue serotype, pre-existing antibodies facilitate the entry of the complex antibody-heterologous virus into target cells, thereby enhancing the infection. This disease augmentation phenomenon is referred to as ADE, posing a significant challenge in developing and popularizing the Dengue virus vaccine [12,13].

In recent years, mathematical modeling has emerged as an essential tool for comprehending infectious disease epidemiology and dynamics at macroscopic and microscopic levels, elucidating ideas about the components of host–pathogen interactions. Dengue models are frequently employed to comprehend infectious disease dynamics and evaluate the effectiveness of intervention strategies such as vector control and vaccination [14,15,16]. In this context, numerous mathematical approaches have been undertaken to investigate host–virus interactions, particularly with respect to virus clearance aided by antibodies [17,18,19,20]. Two notable models are reviewed in this section. The virus–antibody interaction model proposed by Clapham’s group in 2016 [17] quantitatively elucidates virus clearance under antibody assistance. The other model, proposed by Soewono’s group in 2021, seeks to clarify the ADE effect by investigating host–virus interactions [18].

The model proposed by Clapham’s group [17] is summarized as follows. This model, similar to those used for influenza [21,22], describes the interaction between target cells (*x*) and the free virus (*v*) that results in infected cells (*y*) which can produce more viruses. During this process, antibody levels (*z*) increase with the objective of halting the infection and providing protection against subsequent infections. The model is defined by the following equations.

(1) $$\frac{dx}{dt}=A-\gamma x-\beta xv$$

(2) $$\frac{dy}{dt}=\beta xv-\delta y-\alpha zy$$

(3) $$\frac{dv}{dt}=\omega y-\kappa v-\varepsilon zv$$

(4) $$\frac{dz}{dt}=\frac{\eta yz}{\psi +y}$$

This model demonstrates robust fitting performance but possesses several limitations. One notable concern is the inclusion of the *βxv* term to describe the transformation of susceptible cells into infected cells. The immediate consumption of susceptible cells by a substantial viral load caused by this term would rapidly deplete the susceptible cell population, terminating the infection due to cell depletion rather than immune response activation. However, in actual infection cases, infected cells contribute only a small fraction to the overall susceptible cell population. The primary driving force behind virus clearance is the activation of antibodies [23]. Another critical limitation is the mathematical formulation of antibody dynamics as represented in Equation (4). The dynamics of antibodies do not conform to a Michaelis–Menten equation. While this model can capture the dynamics of IgM and IgG in secondary Dengue virus infections, it fails to explain the significant difference in antibody dynamics between primary and secondary infections.

In response to these limitations, Soewono’s group developed a new set of mathematical equations to describe host–virus interactions in Dengue infection [18]. This model explicitly distinguishes between two types of antibodies: IgM and IgG. A concise description of these equations is provided below:

(5) $$\frac{dS}{dt}=\pi_{S}-aSV-\mu_{S}S$$

(6) $$\frac{dI}{dt}=aSV-{(\mu }_{S}+\mu_{i})I$$

(7) $$\frac{dV}{dt}={\kappa \mu }_{i}I-bSV-b_{m}S_{m}V-d_{M}VM-d_{G}VG$$

(8) $$\frac{dS_{m}}{dt}=\pi_{m}-a_{m}S_{m}V-\mu_{S}S_{m}$$

(9) $$\frac{dP}{dt}=\;a_{m}S_{m}V-{(\mu }_{S}+\mu_{P})P$$

(10) $$\frac{dM}{dt}=\;\alpha_{M}P-\gamma_{M}MV-\mu_{M}M$$

(11) $$\frac{dG}{dt}=\alpha_{G}P-\gamma_{G}GV-\mu_{G}G$$

(12) $$\frac{dC_{M}}{dt}=\gamma_{M}MV-\mu_{C_{M}}C_{M}$$

(13) $$\frac{dC_{G}}{dt}=\gamma_{G}GV-\mu_{C_{G}}C_{G}$$

*S* represents susceptible cells, *I* represents infected cells, *V* represents the free virus, *S_m_* represents macrophage cells, *P* represents antigen-presenting cells (APC), *G* represents IgG, *M* represents IgM, $C_{M}$ represents the IgM–virus complex, and $C_{G}$ represents the IgG–virus complex. While this model offers a quantitative explanation of antibody-dependent enhancement, it also presents several hypothetical aspects. Firstly, similar to the βxv term in the previous model, the application of the *aSV* term may lead to a rapid depletion of susceptible cells. Additionally, the inclusion of macrophage cells and antigen-presenting cells raises concerns. The regeneration of neutralizing antibodies is explicitly associated with the concentration of antigen–antibody complexes rather than the antibody level. The primary role of macrophages is to eliminate infected cells with the assistance of neutralizing antibodies, rather than presenting antibodies to antigen-presenting cells [24].

Considering the merits and limitations of these models, along with our prior research on antibody dynamics [25], we propose a novel mathematical model to elucidate Dengue virus–host interactions in this study. This model possesses two key advantages: Firstly, by introducing a smoother term, we successfully eliminate the risk of susceptible cell depletion. Consequently, the termination of infection primarily stems from virus clearance aided by antibodies, with only a minor contribution from the consumption of susceptible cells. Secondly, we incorporate a well-grounded equation to describe the activation effect of the virus on antibody regeneration. In addition to its satisfactory fitting performance, this mathematical model is supported by solid physical foundations, thus enhancing its predictive capability. Ultimately, our model provides an explanation for the significant disparities in IgG and IgM dynamics observed between primary and secondary infections. It facilitates the prediction of the duration of protection against homogeneous infection following a secondary infection and offers a quantitative understanding of antibody-dependent enhancement in heterogeneous infections.

## 2. Materials and Methods

### 2.1. An Overview of Dengue Virus Infection

**Figure 1:**
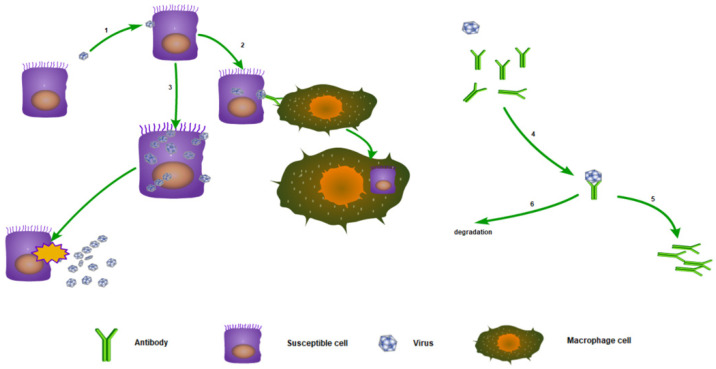
Schematic in-host Dengue immunological responses mediated by antibodies.

Figure 1 depicts the simplified interaction among target cells, infected cells, virus, and immunological response mediated by antibodies. Process (1) entails the viral entry into susceptible cells, representing the transformation of susceptible cells into infected cells. Process (2) involves the clearance of infected cells by natural killer cells and macrophages, aided by the specific binding of antibodies to extramembrane viruses [26]. Process (3) signifies the lysis of infected cells, which occurs as the virus proliferates to a certain threshold. Subsequently, upon cell lysis, numerous free viruses are released into the body. Process (4) denotes the binding between viruses and antibodies, whereby the antibodies exert their neutralizing effect. Process (5) depicts the stimulation of antibody regeneration due to the presence of virus–antibody complexes. Notably, neither viruses nor antibodies directly stimulate antibody reproduction. Instead, the virus–antibody complexes implement positive feedback regulation on antibody regeneration. Consequently, specific neutralizing antibodies with robust binding affinity are selectively produced after infection. While Process (5) offers a basic illustration of antibody regeneration, the underlying mechanisms are considerably more intricate.

In the field of immunology, these virus–antibody complexes localize on the surface of B-cells, since antibodies are initially synthesized by B-cells and bind to the plasma membrane of B-cells. Furthermore, these complexes subsequently interact with helper cells, as antibodies possess another structure that enables them to bind to receptors on these cells. The helper cells present the antigen portion (which, in this case, is the virus) to T-cells. The physical arrangement involves B-cells binding to helper cells and positioning themselves in close proximity to T-cells. T-cells then process these antigenic substances, and if they are non-self, they secrete signaling molecules to facilitate the proliferation or division of B-cells associated with them. Thus, the antibodies produced by these B-cells undergo proliferation [27]. Finally, Process (6) signifies the degradation of virus–antibody complexes, which can be recognized and rapidly degraded by functional immune cells like natural killer cells [28].

In summary, our model relies on several important assumptions. The first assumption is that B cells play a predominant role in antigen presentation during viral infections. Antibodies with high affinity for antigenic substances efficiently bind to them, presenting them to Th cells, which then form germinal centers. Germinal center Th cells promote the proliferation of B cells through the secretion of cytokines, thereby achieving the proliferation of specific antibodies. This process is explicitly represented in our model as Process (5). The second assumption is that we explicitly consider the process of antibody-dependent cellular cytotoxicity (ADCC), which is represented by Process (2). This ADCC effect may be due to the complement system’s killing effect mediated by antibodies or the phagocytic effect of immune cells such as NK cells mediated by antibodies. In addition, in our Model (2), we take into account the conversion between different antibody isotypes, specifically the conversion of IgM to IgG. This conversion is essential for determining whether a patient is experiencing their first infection, because specific IgG in the human body often originates from the conversion of the IgM antibody pool. Therefore, after the first infection, IgM levels increase rapidly, while IgG levels often increase only minimally. However, secondary infections can significantly elevate IgG levels, thus providing more durable immune protection.

### 2.2. Data Resource

We use data obtained in a mathematical modeling work of Dengue virus [17], which contains the virus loading, IgM and IgG titer information from 64 patients. Invitations were extended to adult male individuals seeking medical care at the outpatient department of the Hospital for Tropical Diseases in Ho Chi Minh City, Vietnam. These individuals were suspected of having Dengue fever, which was subsequently confirmed by a positive NS1 rapid test (NS1 STRIP, Bio-Rad). Eligibility for study participation included the following criteria: (1) males between the ages of 18 and 65, (2) a history or presence of fever (temperature ≥ 38 °C) accompanied by clinical suspicion of DENV infection and a positive NS1 rapid test result, (3) symptom onset within the 48 h period prior to initial dosing, and (4) a body mass index ranging from 18 to 35. Patients were enrolled within 48 h of fever onset. The trial had all four subtypes of Dengue virus. 

The concentrations of IgM and IgG antibodies were quantified using the ELISA method at 12 hour intervals, in conjunction with qPCR assessment of viral load in the blood. Within the low antibody concentration range (optical density less than 25), the ELISA method demonstrated a strong linear positive correlation with actual antibody concentrations.

Based on the concentrations and proportions of IgM and IgG, we were able to distinguish between primary and secondary infections. Primary infections are often characterized by higher levels of IgM and lower levels of IgG. Using a threshold of 10 as the IgG titer, we determined that patients 8, 20, 38, 45, 48, 58, and 63 experienced a primary infection, while the remaining patients encountered a secondary infection.

### 2.3. Sensitivity Analysis

The sensitivity analysis followed the approach of [29], in which parameters were varied by one order of magnitude above and below their nominal values. A sensitivity metric, $s_{i,j}$, was then quantified by Equation (14), in which the partial derivative of the output $y_{j}$ with respect to parameter *p_i_* (i.e., a reaction rate constant) was normalized by the nominal values of *p_i_* and $y_{j}$ (i.e., $p_{i}^{0}$ and $y_{j}^{0}$ respectively);

(14) $$s_{i,j}=\frac{p_{i}^{0}dy_{j}}{y_{j}^{0}dp_{i}}|p_{0}$$

where the vector $p_{0}$ is a vector of nominal values of all parameters in the model. In this work, the output of the system, i.e., $y_{j\;}$ in Equation (11), was set to the maximal value of the production rate of virus, IgM, and IgG, respectively for *j* equal to 1, 2, and 3.

## 3. Results

### 3.1. A Simple Model without Antibodies’ Classification (Model 1)

We expanded our antibody dynamics model to include the susceptible cell transformation. A simple model without antibodies’ classification is presented below:

(15) $$\frac{dS}{dt}=\pi_{S}-aS\frac{V}{V+K_{m}}-\mu S$$

(16) $$\frac{dI}{dt}=aS\frac{V}{V+K_{m}}-\beta I\frac{G}{G+K_{m}\prime }-\gamma I$$

(17) $$\frac{dV}{dt}=\kappa \gamma I-\theta VG+\xi C_{G}$$

(18) $$\frac{dG}{dt}=-\theta VG+\xi C_{G}+\eta C_{G}-\rho G$$

(19) $$\frac{dC_{G}}{dt}=\;\theta VG-\lambda C_{G}$$

*S* represents the susceptible cell (*S*); *I* represents the infected cell (*I*); *V* represents the free virus (*V*); *G* represents the antibody (*G*); and $C_{G}$ represents the virus–antibody complex ($C_{G}$). Susceptible target cells (*S*) are continually produced by the body at a constant rate (*πS*) and have a natural mortality rate *µS*, where 1/μS represents the expected lifetime of an uninfected, i.e., susceptible, target cell. Unlike classical models, we employ $aS\frac{V}{V+K_{m}}$ instead of *aSV* to represent the susceptible–infected transformation, thereby effectively preventing the depletion of susceptible cells. The transformation from susceptible cells to infected cells corresponds to Process (1) in Figure 1. In Equation (15), $\beta I\frac{G}{G+K_{m}\prime }$ represents the clearance of infected cells by macrophages aided by antibodies, with *β* denoting the maximal clearance rate that can be achieved at a high antibody level. This term corresponds to Process (2) in Figure 1. Unlike the self-lysis described in Process (3) [27], the phagocytosis of susceptible cells by macrophages does not release free virus into the host body. γI signifies the self-lysis of infected cells, whereby, as viruses proliferate within their host cells, infected cells eventually lyse if not cleared by macrophages. γ denotes the rate of self-lysis, resulting in the release of κγI viruses into the body environment. This process corresponds to Process (3) in Figure 1. The term θVG in the equation represents the binding of antibodies with viruses, corresponding to Process (4). $\xi C_{G}$ represents the dissociation of virus–antibody complexes, which is the reverse reaction of the binding process. *ξ* denotes the dissociation constant, which is generally very small and can be neglected in the simulation [30]. $\eta C_{G}$ represents antibody regeneration activated by the presence of virus–antibody complexes, corresponding to Process (5) in Figure 1. $\lambda C_{G}$ represents the clearance of virus–antibody complexes with rate constant λ, corresponding to Process (6). ρG represents the degradation of antibodies with rate ρ.

**Figure 2:**
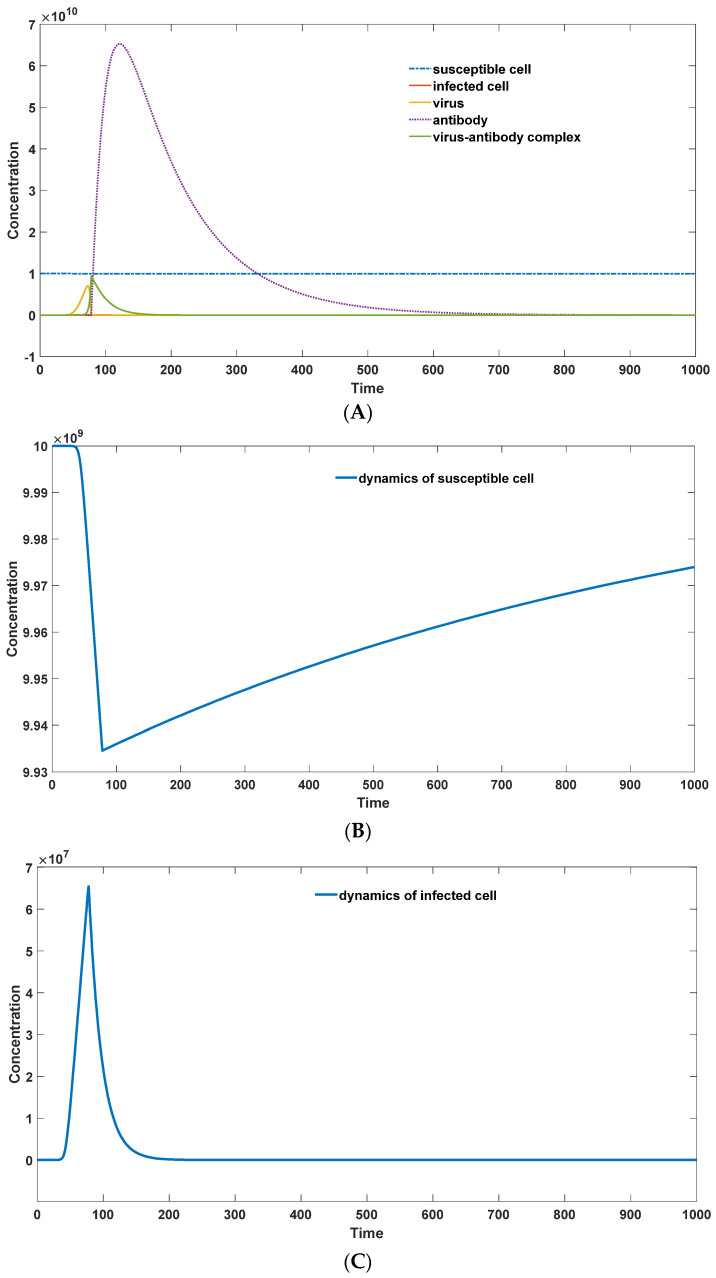
(**A**): The dynamics of all components during the infection. (Parameter set: *S*_0_ = 1 × 10^10^; *I*_0_ = 0; *V*_0_ = 1; *G*_0_ = 1 × 10^1^; $C_{G}$_0_ = 0; π = 1 × 10^7^; *μ* = 1 × 10^−3^; a = 2 × 10^−4^; β = 5 × 10^−2^; γ = 1 × 10^−5^; κ = 1 × 10^6^; ξ = 1 × 10^−14^; *θ* = 1 × 10^−5^; ρ = 0.01; *η* = 0.5; *λ* = 1 × 10^−1^; $K_{m}$ = 1 × 10^8^; $K_{m_{1}}\prime$ = 1 × 10^6^); (**B**): the dynamics of susceptible cells in the simulation; (**C**): the dynamics of infected cells in the simulation.

The simulation results of the infection mediated by antibodies are represented in Figure 2. In Figure 2A, the efficient elimination of the virus occurs after antibody proliferation. An interesting phenomenon is observed whereby the virus (solid yellow line) increases at a faster rate than the virus–antibody complexes (solid green line). Immune responses are directly correlated with the virus–antibody complexes. Therefore, patients remain asymptomatic during the initial days of infection, even though their virus loads reach relatively high levels [17,31]. Symptoms manifest when the virus–antibody complexes reach a certain threshold, at which point the virus antibody level is consistently near or beyond its peak. This observation can explain why the virus load is always maximal when symptoms first appear in Dengue virus infection [31,32]. The virus load begins to decline after symptom onset. Additionally, it is noted in Figure 2A that the concentration of free-neutralizing antibodies starts to increase much later than the virus–antibody complexes. The antibodies generated earlier bind to viruses to form virus–antibody complexes. ELISA tests provide the concentration of the overall antibody level, encompassing both free and bound states [33]. One advantage of this model is the prevention of susceptible cell depletion. As shown in Figure 2B,C, infected cells only constitute a small fraction of the total susceptible cells, and the susceptible cell count returns to normal levels after infection.

### 3.2. A Mathematical Model with Antibodies’ Classification (Model 2)

A mathematical model with different antibody types is further developed to simulate better the divergent behaviors of different antibodies (IgM and IgG in this case). A set of equations is displayed below:

(20) $$\frac{dS}{dt}=\pi_{S}-aS\frac{V}{V+K_{m}}-\mu S$$

(21) $$\frac{dI}{dt}=aS\frac{V}{V+K_{m}}-\beta I\frac{G}{G+K_{m}\prime }-\beta I\frac{M}{M+K_{m}\prime }-\gamma I$$

(22) $$\frac{dV}{dt}=\kappa \gamma I-\theta VG-\theta VM$$

(23) $$\frac{dG}{dt}=-\theta VG+\eta C_{G}-\delta G+\epsilon M$$

(24) $$\frac{dC_{G}}{dt}=\;\theta VG-\lambda C_{G}$$

(25) $$\frac{dM}{dt}=-\theta VM+\eta C_{M}-\chi M-\epsilon M$$

(26) $$\frac{dC_{M}}{dt}=\theta VM-\lambda C_{M}$$

The term $\beta I\frac{G}{G+K_{m}\prime }$ represents the cytotoxic effect of natural killer (NK) cells on infected cells through antibody-dependent cellular cytotoxicity (ADCC) facilitated by IgG. Similarly, the term $\beta I\frac{M}{M+K_{m}\prime }$ represents the cytotoxic effect of NK cells on infected cells through ADCC facilitated by IgM. As IgG is derived from the conversion of IgM isotypes, the rate of this conversion is denoted by *ϵ*, and both IgG and IgM have the same binding coefficient *θ* with the virus. They stimulate the further proliferation of antibodies through the antibody–virus complex formed with the same positive feedback coefficient *η*. *δ* represents the decay constant of IgG. Since IgM decays faster than IgG, a larger factor *χ* is added to represent the decay rate (*χM*) of IgM. *λ* represents the clearance rate of the antibody–virus complex.

**Figure 3:**
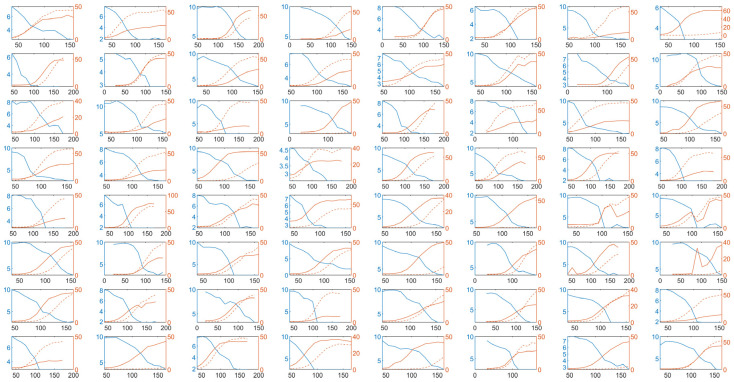
Virus–antibody dynamics of 64 Dengue virus infections. The x-axis represents time since the onset of symptoms, in hours (h). The left y-axis represents log (viral load), while the right y-axis represents antibody titers. The solid blue line represents viral load, the solid red line represents changes in IgM concentration, and the dashed red line represents changes in IgG concentration.

The clinical data of 64 patients with different types of Dengue fever are shown in Figure 3.

From Figure 3, we can observe two interesting phenomena. The first one is that the ratio of IgM to IgG can distinguish whether a patient is experiencing a primary infection or a secondary infection. In patients with a primary infection, the proportion of IgG is low and there is no significant increase in IgG levels. However, during a secondary infection, both IgM and IgG levels show a significant increase. From Figure 3, it can be observed that patients 8, 20, 38, 45, 48, 58, and 63 experienced a primary infection, as their IgG levels did not show a significant increase following the infection. The second interesting phenomenon is that although there is a significant difference in peak viral load among different patients, with some patients having incomplete clinical data making it difficult to determine the maximum viral load, there are also patients who can confidently determine the peak viral load during their infection period. The difference in peak viral load can exceed 100-fold, yet all patients reach a similar level of maximum antibody production. Our model can explain these two phenomena effectively.

**Figure 4:**
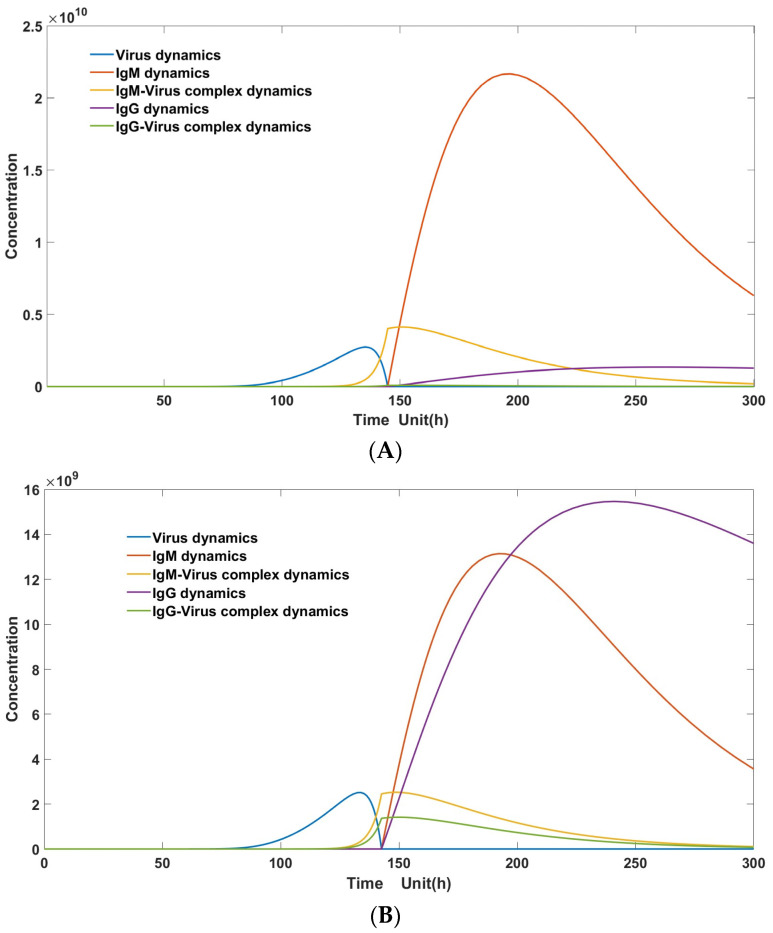
(**A**): Model simulation: primary Dengue infection immunological responses mediated by antibodies. (Parameter set: *S*_0_ = 1 × 10^10^; *I*_0_ = 0; *V*_0_ = 1; *M*_0_ = 1 × 10^1^; $C_{M}$_0_ = 0; *G*_0_ = 0; $C_{G}$_0_ = 0; π = 1 × 10^7^; μ = 0.5 × 10^−3^; a = 1 × 10^−4^; β = 2.5 × 10^−2^; γ = 0.5 × 10^−5^; κ = 0.5 × 10^6^; θ = 1 × 10^−5^; δ = 0.005; η = 0.25; λ = 0.5 × 10^−1^; $K_{m}$ = 0.5 × 10^8^; $K_{m}\prime$ = 0.5 × 10^6^; χ = 0.025); (**B**): model simulation: secondary Dengue infection immunological responses mediated by antibodies. (Parameter set: *S*_0_ = 1 × 10^10^; *I*_0_ = 0; *V*_0_ = 1; *M*_0_ = 1 × 10^1^; $C_{M}$_0_ = 0; *G*_0_ = 2; $C_{G}$_0_ = 0; *π* = 1 × 10^7^; μ = 0.5 × 10^−3^; a = 1 × 10^−4^; β = 2.5 × 10^−2^; γ = 0.5 × 10^−5^; κ = 0.5 × 10^6^; θ = 1 × 10^−5^; δ = 0.005; η = 0.25; λ = 0.5 × 10^−1^; $K_{m}$ = 0.5 × 10^8^; $K_{m}\prime$ = 0.5 × 10^6^; χ = 0.025).

During the initial infection, since there are no B cells producing IgG present in the body, the initial concentration of IgG is zero. IgG is entirely derived from the conversion of B cells producing IgM to those producing IgG. Therefore, during the first infection, the level of IgG does not rise to a high level due to the abundant production of IgM, which leads to the complete clearance of the virus. IgG ceases to proliferate as it loses stimulation from antigen–antibody complexes, resulting in its level being maintained at a relatively low state, as depicted in Figure 4A. However, during the second infection, the initial concentration of IgG is non-zero, so its growth mainly comes from the stimulus of IgG–virus complexes for its renewed production, rather than primarily from the conversion of IgM. As a result, both IgG and IgM levels rise to a comparatively high level, as illustrated in Figure 4B.

To elucidate the second phenomenon, we conducted a parameter sensitivity analysis on our model, aiming to identify the crucial parameter that exhibits a significant impact on the peak viral load while having minimal effect on the maximum antibody production.

The results of the parameter sensitivity analysis are presented in Table 1.

**Figure 5:**
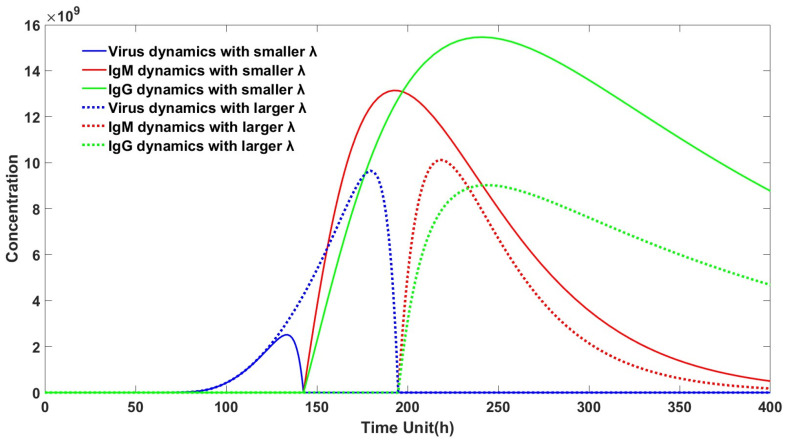
Virus–antibody dynamics at different virus–antibody clearance rates. Infections with slower clearance rates of antibody–virus complexes are marked in solid line. Infections with faster clearance rates of antibody–virus complexes are marked in dash line.

From Table 1, it can be observed that the variation of λ significantly affects the peak viral load, while it does not have a significant impact on the maximum antibody production. Here, λ represents the clearance rate of antibody–virus complexes, and at the cellular level, the clearance of antigen–antibody complexes is primarily mediated by NK cells. From Figure 5, it can be seen that when λ is small, indicating a slower clearance rate of antibody–virus complexes, the peak viral load is small, and the concentration of virus–antibody complexes is low. In such cases, patients often exhibit weaker clinical symptoms, which aligns with our clinical observations. On the other hand, when λ is large, indicating a faster clearance rate of antibody–virus complexes, the peak viral load is high, and the concentration of virus–antibody complexes is high. Consequently, patients tend to exhibit more severe clinical symptoms and longer infection periods. This conclusion appears paradoxical because, traditionally, NK cells have been recognized for their active role in the clearance of antibody–virus complexes and their mediating role in ADCC in adaptive immunity. However, recent reports have consistently revealed a negative correlation between NK cells and humoral immunity. Elevated levels of NK cells and excessive NK cell cytotoxicity can hinder antibody generation and increase the occurrence of severe cases, as confirmed in chronic LCMV infection [34]. Our model provides a sound explanation for this phenomenon. Since NK cells directly participate in the ADCC process, they not only clear infected cells but also eliminate helper T cells bound to B cells (since the surface of the B cell–T cell conjugate complex expresses antibodies). Thus, this clearance of antigen–antibody complexes leads to a decrease in the number of helper T cells, resulting in a delayed antibody regeneration process. Consequently, this delay in humoral immunity leads to higher peak viral load and a greater concentration of virus–antibody complexes, ultimately contributing to the occurrence of more severe clinical symptoms. Therefore, reducing the level or cytotoxic activity of NK cells may play a certain positive role in preventing the development of severe cases.

In terms of setting the initial parameters, we did not use traditional parameter-fitting methods [35]. Instead, we evaluated the reliability of our parameters using several key indicators: peak viral load concentration and its appearance time, and peak antibody concentration and its peak concentration appearance time. The reasons for not using parameter-fitting methods for parameter estimation are as follows:

1. There are a large number of parameters involved, and the accuracy of fitting may be affected by using parameter-fitting methods;
2. There are significant fluctuations in the experimental data on a logarithmic scale, especially in viral load, ranging from several hundred to 10^10^. If using the minimum variance between simulated and experimental data as the objective function for optimization, it would neglect those time points with lower concentrations. Fitting after logarithmic transformation would weaken the weight of high-concentration sites;
3. Experimental data cannot effectively represent the true concentration of various substances. For example, changes in viral load measured in experiments include the concentration of free viruses and a portion of the virus binding to antibodies. At the same time, the measurement of antibody concentration is not the absolute concentration of unbound antibodies;
4. It is impossible to effectively calculate the time points. Experimental data can only reflect the changes in the concentration of each substance from the onset of disease, rather than from the onset of infection. Because the initial infectious dose may vary greatly, the incubation period may also vary greatly, making it impossible to effectively calculate the time points.

Considering the aforementioned reasons, qualitative analysis using experimental data is more reliable than simple quantitative calculations. The significance of employing mathematical models lies in exploring potential underlying mechanisms rather than fitting to known data. Our model reflects that, for different individuals, the properties of antibodies, including their ability to bind to the virus and their decay periods, may not exhibit significant differences when facing Dengue virus infection. Thus, a crucial factor contributing to individual variations in infection may be the disparities in the quantity and subtype of NK cells, which can result in differences in the clearance rates of antigen–antibody complexes. The presence of highly active NK cell functionality could potentially contribute to severe infections.

Given these parameter values, we can predict the protection threshold of IgG in avoiding homogenous reinfection. This threshold is calculated to be around 1 × 10^6^. Reinfection could happen once the IgG level drops below this threshold. If the degradation of IgG follows the −*δG* term, we could also calculate the protection duration (about 130 days in this case). However, the actual antibody decay did not obey this simple rule. IgG decays at a lower rate as time increases [36]. This can be explained in our antibody dynamics theory, in which a new term named “environmental antigens” is introduced. A more complicated model is represented here when we consider environmental antigens’ function in slowing antibodies’ decay rates.

(27) $$\frac{dS}{dt}=\pi_{S}-aS\frac{V}{V+K_{m}}-\mu S$$

(28) $$\frac{dI}{dt}=aS\frac{V}{V+K_{m}}-\beta I\frac{G}{G+K_{m}\prime }-\beta I\frac{M}{M+K_{m}\prime }-\gamma I$$

(29) $$\frac{dV}{dt}=\kappa \gamma I-\theta VG-\theta VM$$

(30) $$\frac{dG}{dt}=-\theta VG+\eta C_{G}-\omega EG+\eta C_{EG}-\delta G+\epsilon M$$

(31) $$\frac{dC_{G}}{dt}=\theta VG-\lambda C_{G}$$

(32) $$\frac{dM}{dt}=-\theta VM+\eta C_{G}-\omega EM+\eta C_{EM}-\chi M-\epsilon M$$

(33) $$\frac{dC_{M}}{dt}=\theta VM-\lambda C_{M}$$

(34) $$\frac{dE}{dt}=0$$

(35) $$\frac{dC_{EG}}{dt}=\;\omega EG-\lambda C_{EG}$$

(36) $$\frac{dC_{EM}}{dt}=\;\omega EM-\lambda C_{EM}$$

*E* represents environmental antigens. It would remain at a very stable level due to a rapid replenishment from the environment. $C_{EG}$ is the environmental antigen–IgG complex. $C_{EM}$ is the environmental antigen–IgM complex. The environmental antigens would bind IgG with a binding rate ω and IgM with a binding constant ω. If we can find the clinical data of IgG dynamics in a relatively long time, we could estimate the level of environmental antigens *E*, ω. The antibody decay would no longer follow a simple term −δG. The calculated protection time would be much longer than that deferred in the second model. Unluckily, we did not find a long-term IgG dynamic in Dengue infection in this study.

### 3.3. A Mathematical Model Simulating Antibody-Dependent Enhancement (ADE) (Model 3)

As introduced in the introduction, a very interesting phenomenon of Dengue virus infection is the ADE effect after a heterogeneous infection. To simulate the ADE effect, a modified mathematical model is represented below:

(37) $$\frac{dS}{dt}=\pi_{S}-f\left(a\right)S\frac{V}{V+K_{m}}-\mu S$$

(38) $$\frac{dI}{dt}=f\left(a\right)*S\frac{V}{V+K_{m}}-\beta I\frac{G_{1}}{G_{1}+K_{m_{1}}\prime }-\beta I\frac{G_{2}}{G_{2}+K_{m_{2}}\prime }-\gamma I$$

(39) $$\frac{dV}{dt}=\kappa \gamma I-\theta_{1}VG_{1}-\theta_{2}VG_{1}$$

(40) $$\frac{dG_{1}}{dt}=-\theta_{1}VG_{1}+\eta C_{G_{1}}-\delta G_{1}$$

(41) $$\frac{dC_{G_{1}}}{dt}=\theta_{1}VG_{1}-\lambda C_{G_{1}}$$

(42) $$\frac{dG_{2}}{dt}=-\theta_{2}VG_{1}+\eta C_{G_{2}}-\delta G_{2}$$

(43) $$\frac{dC_{G_{2}}}{dt}=\theta_{2}VG_{2}-\lambda C_{G_{2}}$$

(44) $$f\left(a\right)=a(\varepsilon \frac{C_{G_{2}}}{C_{G_{2}}+V}+1)$$

$G_{2}$ represents the IgG antibodies associated with antibody-dependent enhancement (ADE), which exhibit a specific elevation level following the initial infection. Conversely, $G_{1}$ represents a novel subtype-specific IgG response developed against the new Dengue virus strain. $G_{1}$ demonstrates a superior binding affinity $\theta_{1}$ towards the new virus subtype, while $G_{2}$ exhibits relatively lower binding affinity $\theta_{2}$. Consequently, the clearance efficiencies of infected cells mediated by these two types of IgG differ. This discrepancy can be attributed to the variance in virus-binding capabilities. $K_{m_{1}}\prime$ is smaller than $K_{m_{2}}\prime$, owing to a stronger binding affinity. $C_{G_{2}}$ represents the virus–$G_{2}$ complex, while $C_{G_{1}}$ represents the virus–$G_{1}$ complex.

**Figure 6:**
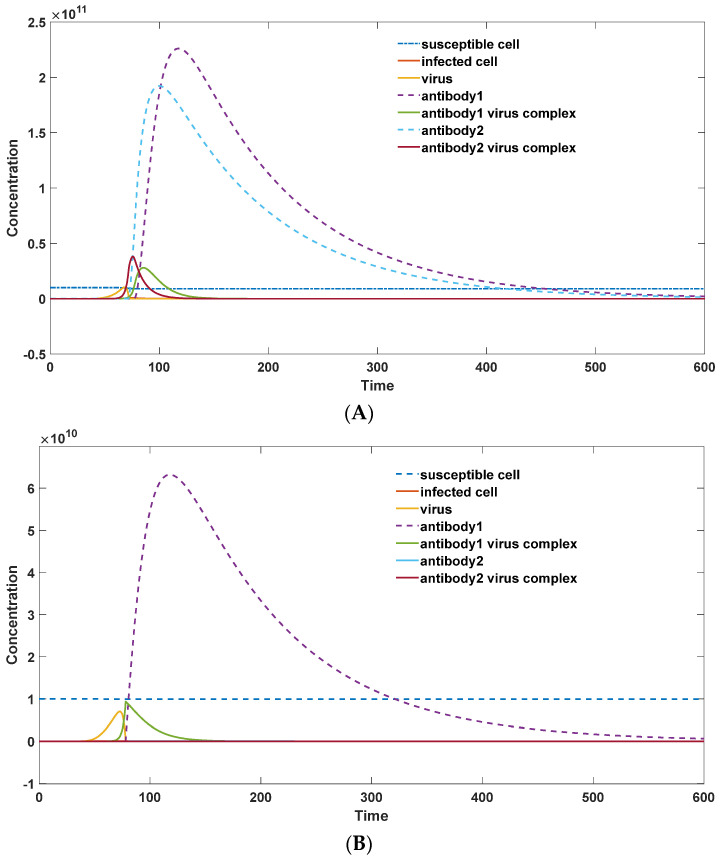
(**A**): Modeling of ADE in the presence of weakly binding antibodies (parameter set: *S*_0_ = 1 × 10^10^; *I*_0_ = 0; *V*_0_ = 1; $G_{1}$_0_ = 1 × 10^1^; $C_{G_{1}}$_0_ = 0; $G_{2}$_0_ = 1 × 10^5^; $C_{G_{2}}$_0_ = 0; π = 1 × 10^7^; μ = 1 × 10^−3^; a = 2 × 10^−4^; β = 5 × 10^−2^; γ = 1 × 10^−5^; κ = 1 × 10^6^; ε = 1 × 10^2^; $\theta_{1}$ = 1 × 10^−5^; $\theta_{2}$ = 1 × 10^−8^; δ = 0.01; η = 0.5; λ = 1 × 10^−1^; $K_{m}$ = 1 × 10^8^; $K_{m_{1}}\prime$ = 1 × 10^6^; $K_{m_{2}}\prime$ = 1 × 10^8^); (**B**): virus–host interaction in the low concentration of weakly binding antibodies in primary infection (parameter set: *S*_0_ = 1 × 10^10^; *I*_0_ = 0; *V*_0_ = 1; $G_{1}$_0_ = 1 × 10^1^; $C_{G_{1}}$_0_ = 0; $G_{2}$_0_ = 1 × 10^1^; $C_{G_{2}}$_0_ = 0; π = 1 × 10^7^; μ = 1 × 10^−3^; a = 2 × 10^−4^; β = 5 × 10^−2^; γ = 1 × 10^−5^; κ = 1 × 10^6^; ε = 1 × 10^2^; $\theta_{1}$ = 1 × 10^−5^; $\theta_{2}$ = 1 × 10^−8^; δ = 0.01; η = 0.5; λ = 1 × 10^−1^; $K_{m}$ = 1 × 10^8^; $K_{m_{1}}\prime$ = 1 × 10^6^; $K_{m_{2}}\prime$ = 1 × 10^8^).

$f\left(a\right)$ symbolizes the antibody-dependent enhancement resulting from the presence of $G_{2}$. $G_{2}\;$ acts to neutralize the virus while concurrently promoting the formation of infected cells. When a heterogenous secondary infection occurs, the initial concentration of $G_{2}$ surpasses that of $G_{1}$ due to its elevation resulting from the primary infection. During the early stages of heterogenous infection, $G_{2}$ can bind with viruses to generate a substantial number of virus–$G_{2}$ complexes. These $C_{G_{2}}$ complexes facilitate virus entry into susceptible cells through a scaling factor $\left(\varepsilon \frac{C_{G_{2}}}{C_{G_{2}}+V}+1\right)$. As the concentration of $C_{G_{2}}$ decreases significantly, the ADE effect diminishes, and the scaling factor becomes equal to one ($f\left(a\right)=a$). Conversely, a maximal ADE effect can be achieved when $C_{G_{2}}$ greatly outweighs the virus concentration ($f\left(a\right)=a(\varepsilon +1)$). The modeling results pertaining to ADE are illustrated in Figure 6.

As illustrated in Figure 6, the peak virus load (represented by the solid yellow line in Figure 6A) during heterogeneous infection surpasses the corresponding load (solid yellow line in Figure 6B) observed in primary infection. Furthermore, the antibody–virus complexes (depicted as a combination of solid green and purple lines in Figure 6A) formed during heterogeneous infection are significantly larger than those (depicted as a combination of solid green and purple lines in Figure 6B) observed in primary infection. This disparity could lead to more severe infection symptoms during heterogenous secondary infections. Moreover, it is worth noting that both $G_{2}$ and $G_{1}$ exhibit heightened levels compared to primary infection.

## 4. Discussion

Mathematical models offer a quantitative assessment of the dynamics of host–virus interactions. The application of mathematical modeling in studying immunological responses to Dengue fever is particularly noteworthy, not only due to its practical implications but also owing to its inherent complexity. Notably, there exists a notable disparity in antibody performance between primary and secondary Dengue infections. Experimental reports indicate that during the initial infection, IgM levels surge while in secondary infection, and IgG exhibits a significant increase. Additionally, Dengue fever is characterized by antibody-dependent enhancement, which renders heterogenous secondary infections more fatal than preceding infections. To elucidate these phenomena, we have developed a novel mathematical model.

Compared to previous research, our study has several key improvements. Firstly, when describing the process of viral infection in susceptible cells, we avoided using the *αSV* term, which is a classic model based on second-order chemical reaction kinetics. However, this model assumes a one-to-one binding relationship between the virus and the cell, which is not the case in real-life infections where multiple viruses can infect a single cell. Previous computational biologists have recognized that the target cell limitation model fails to explain viral dynamics in such cases [37]. Assuming the probability of a single virus infecting a cell is *α*, the average number of infected cells after *V* viruses infect *S* cells is *S* (1 − (1 − *α*)*V*), which differs significantly from the *αSV* model, especially when *V* is large. Using the Michaelis–Menten equation to represent the rate of virus infecting susceptible cells as $aS\frac{V}{V+K_{m}}$ can avoid the phenomenon of target-cell depletion. The second improvement is the use of the Michaelis–Menten equation in the form of $\beta I\frac{G}{G+K_{m}\prime }$ to represent ADCC effects, indicating that antibody production greatly accelerates the clearance of infected cells. Avoiding the use of the term βIG also better conforms to the dynamic characteristics of ADCC. The third major improvement is that our model avoids using fitted mathematical formulas to represent the virus’s counteracting effect on antibodies. Many modeling attempts have been made to fit experimental data, using various mathematical functions and parameters. Increasing the number of compartments and parameters naturally improves fitting performance [38]. As John von Neumann famously quipped, “With four parameters, I can fit an elephant, and with five, I can make him wiggle his trunk”. However, these equations lack substantial physical support, limiting their predictive capabilities. In light of this, we have reformulated the activation effects of the virus on antibody regeneration, directly linking it to the level of virus–antibody complexes, a relationship strongly supported by immunology principles. We explicitly represent this effect through a mathematical formula that provides a better description of the antigen’s stimulating effect on antibodies and explains why antibodies with excellent binding affinity can proliferate rapidly, while those with weak binding affinity are gradually eliminated. Three illustrative schemes (Figure 1) have yielded three representative models. Our model provides a more reasonable explanation for the distinct behaviors of antibodies in primary and secondary infections (Figure 4). Additionally, our model demonstrates good performance in fitting and accurately capturing clinical data. It also allows for the quantitative calculation of the minimal IgG threshold required to prevent reinfection.

Arguably, the most significant finding of this study, from a mathematical modeling perspective, is the discovery of the inhibitory role of natural killer (NK) cells in humoral immunity [34]. The concentration of NK cells or their cytotoxic activity can have contrasting effects. This phenomenon is observed not only in Dengue fever but also in COVID-19 infections, where severe cases are often associated with specific immunotypes of NK cells [39]. Conventionally, this association is attributed to elevated viral load and severe inflammatory responses that contribute to NK cell alterations. However, our research proposes an alternative possibility, suggesting that the severity of infection may be attributed to differences in NK cell subtypes. Highly phagocytic NK cells can engulf and eliminate helper T cells, thereby impeding antibody proliferation and facilitating viral replication. Therefore, severe patients may share certain genetic similarities in their NK cell profiles.

Our model may also help determine the duration of protection by fitting long-term IgG dynamic data. Furthermore, we simulate the antibody-dependent enhancement (ADE) effect using Model 3, shedding light on why heterogenous secondary infections are more fatal than primary infections. We elucidate how non-specific neutralizing IgG antibodies promote secondary infections (Figure 6). These findings, providing insights into the immunopathogenesis of severe diseases caused by pre-existing antibodies and the ADE process, offer valuable contributions to future research assessing the impact of imperfect Dengue vaccines. As we have explicitly included the process of IgM to IgG conversion, we can explain why initial infection does not lead to a rapid increase in IgG levels. This has important implications for vaccine development, suggesting that for Dengue fever vaccines, multiple doses may be required to achieve a significant increase in IgG levels and obtain a relatively long-lasting protective effect. This is similar to the vaccination strategy for COVID-19 vaccines.

Nevertheless, we must acknowledge the limitations of our model, which exist in two main aspects. Firstly, our model cannot replicate the complexity of the human immune system, particularly as it does not directly differentiate between Th cells, B cells, and antibodies, meaning that the interaction process between Th cells and B cells is not explicitly represented. The second main limitation lies in the significant uncertainty present in the data fitting process. As most antibody data is relative, the units are often arbitrary, and there can be considerable numerical differences depending on the method used to measure antibody levels. For example, according to clinical data, IgG peak concentrations measured by multiplex immunoassay can easily exceed 20,000, whereas those measured by standardized ELISA methods are generally within 500. Furthermore, due to the presence of noise, the use of standardized ELISA and other methods for measuring IgG may result in a small initial value even when no specific IgG antibodies are present. Due to these uncertainties, we can often only make relative judgments through numerical fitting, for instance, predicting the antibody protection period of an individual or group, or comparing the strength of NK cell activity between them. However, such comparisons may lose their broad applicability due to changes in the fitted data.

## Figures and Tables

**Table 1:** Sensitivity analysis of parameters against maximal antibody level and peak virus level.

Parameter Name	Sensitivity toward IgM	Sensitivity toward IgG	Sensitivity toward Virus
a	5.505600475	10.15994851	11.0597522
β	−0.341486983	−0.252679972	−0.002039784
γ	5.590870351	10.30647025	11.1861965
κ	5.596633437	10.30563892	11.19329953
θ	−0.29379	−0.19359	−0.33656
η	−0.99562	−0.99605	−0.99979
χ	2.004046	−0.99031	0.228743
λ	−0.3862	−0.15146	5.456431
$K_{m}$	−0.97186	−0.99078	−0.99278
$K_{m}\prime$	0.01285	0.011263	0.000409
δ	−0.74517	0.350095	0.050613
ϵ	0.162458	−0.0943	−0.00385

## Data Availability

Matlab source codes are available at https://github.com/zhaobinxu23/DengueDengue_infection (accessed on 1 January 2024).

## References

[1] Khetarpal N., Khanna I. (2016). Dengue fever: Causes, complications, and vaccine strategies. J. Immunol. Res..

[2] Bhatt S., Gething P.W., Brady O.J., Messina J.P., Farlow A.W., Moyes C.L., Drake J.M., Brownstein J.S., Hoen A.G., Sankoh O. (2013). The global distribution and burden of Dengue. Nature.

[3] Halstead S.B. (2002). Dengue hemorrhagic fever: Two infections and antibody dependent enhancement, a brief history and personal memoir. Rev. Cubana Med. Trop..

[4] Hu D., Di B., Ding X., Wang Y., Chen Y., Pan Y., Wen K., Wang M., Che X. (2011). Kinetics of non-structural protein 1, IgM and IgG antibodies in Dengue type 1 primary infection. Virol. J..

[5] Innis B.L., Nisalak A., Nimmannitya S., Suntayakorn S., Hoke C.H., Chongswasdi V., Puttisri P., Kusalerdchariya S. (1989). An enzyme-linked immunosorbent assay to characterize Dengue infections where Dengue and Japanese encephalitis co-circulate. Am. J. Trop. Med. Hyg..

[6] Prince H.E., Yeh C., Lapé-Nixon M. (2011). Utility of IgM/IgG ratio and IgG avidity for distinguishing primary and secondary Dengue virus infections using sera collected more than 30 days after disease onset. Clin. Vaccine Immunol..

[7] Rothman A.L. (2009). Cellular immunology of sequential Dengue virus infection and its role in disease pathogenesis. Dengue Virus.

[8] St.John A.L., Rathore A.P.S. (2019). Adaptive immune responses to primary and secondary Dengue virus infections. Nat. Rev. Immunol..

[9] Halstead S.B. (2015). Dengue antibody-dependent enhancement: Knowns and unknowns. Antibodies Infect. Dis..

[10] Roehrig J.T. (2003). Antigenic structure of flavivirus proteins. Adv. Virus Res..

[11] Billings L., Fiorillo A., Schwartz I.B. (2008). Vaccinations in disease models with antibody-dependent enhancement. Math. Biosci..

[12] Shukla R., Ramasamy V., Shanmugam R.K., Ahuja R., Khanna N. (2020). Antibody-dependent enhancement: A challenge for developing a safe Dengue vaccine. Front. Cell. Infect. Microbiol..

[13] Miller N. (2010). Recent progress in Dengue vaccine research and development. Curr. Opin. Mol. Ther..

[14] Aguiar M., Stollenwerk N., Halstead S.B. (2016). The impact of the newly licensed Dengue vaccine in endemic countries. PLoS Neglected Trop. Dis..

[15] Aguiar M., Stollenwerk N. (2017). Mathematical models of Dengue fever epidemiology: Multi-strain dynamics, immunological aspects associated to disease severity and vaccines. Commun. Biomath. Sci..

[16] Morales N.L.G., Núñez-López M., Ramos-Castañeda J., Velasco-Hernández J. (2017). Transmission dynamics of two Dengue serotypes with vaccination scenarios. Math. Biosci..

[17] Clapham H.E., Quyen T.H., Kien D.T.H., Dorigatti I., Simmons C.P., Ferguson N.M. (2016). Modelling virus and antibody dynamics during Dengue virus infection suggests a role for antibody in virus clearance. PLoS Comput. Biol..

[18] Sebayang A.A., Fahlena H., Anam V., Knopoff D., Stollenwerk N., Aguiar M., Soewono E. (2021). Modeling Dengue immune responses mediated by antibodies: A qualitative study. Biology.

[19] Nuraini N., Tasman H., Soewono E., Sidarto K.A. (2009). A with-in host Dengue infection model with immune response. Math. Comput. Model..

[20] Ansari H., Hesaaraki M. (2013). A within-host Dengue infection model with immune response and nonlinear incidence rate. Appl. Math..

[21] Smith A.M., Perelson A.S. (2011). Influenza A virus infection kinetics: Quantitative data and models. Wiley Interdiscip. Rev. Syst. Biol. Med..

[22] Parrino J., Graham B.S. (2006). Smallpox vaccines: Past, present, and future. J. Allergy Clin. Immunol..

[23] Wahala W.M.P.B., De Silva A.M. (2011). The human antibody response to Dengue virus infection. Viruses.

[24] Gordon S. (2007). The macrophage: Past, present and future. Eur. J. Immunol..

[25] Xu Z., Yang D., Zhang H. (2021). Antibody Dynamics Simulation-Theory and Application. Research.

[26] Nowak M., May R.M. (2000). Virus Dynamics: Mathematical Principles of Immunology and Virology: Mathematical Principles of Immunology and Virology.

[27] Owen J.A., Punt J., Stranford S.A., Jones P., Owen J. (2013). Kuby Immunology.

[28] Perrin L.H., Oldstone M.B.A. (1977). The formation and fate of virus antigen-antibody complexes. J. Immunol..

[29] Xu Z., Karlsson J.O.M., Huang Z. (2015). Modeling the dynamics of acute phase protein expression in human hepatoma cells stimulated by IL-6. Processes.

[30] Burton D.R., Williamson R.A., Parren P.W.H.I. (2000). Antibody and virus: Binding and neutralization. Virology.

[31] Nguyen N.M., Tran C.N.B., Phung L.K., Duong K.T.H., Huynh H.l.A., Farrar J., Nguyen Q.T.H., Tran H.T., Nguyen C.V.V., Merson L. (2013). A randomized, double-blind placebo controlled trial of balapiravir, a polymerase inhibitor, in adult Dengue patients. J. Infect. Dis..

[32] Tricou V., Minh N.N., Van T.P., Lee S.J., Farrar J., Wills B., Tran H.T., Simmons C.P. (2010). A randomized controlled trial of chloroquine for the treatment of Dengue in Vietnamese adults. PLoS Neglected Trop. Dis..

[33] Chau T.N.B., Hieu N.T., Anders K.L., Wolbers M., Lien L.B., Hieu L.T.M., Hien T.T., Hung N.T., Farrar J., Whitehead S. (2009). Dengue virus infections and maternal antibody decay in a prospective birth cohort study of Vietnamese infants. J. Infect. Dis..

[34] Cook K.D., Kline H.C., Whitmire J.K. (2015). NK cells inhibit humoral immunity by reducing the abundance of CD4+ T follicular helper cells during a chronic virus infection. J. Leucoc. Biol..

[35] Geletu A. (2007). Solving Optimization Problems Using the Matlab Optimization Toolbox—A Tutorial.

[36] Sakhi H., Dahmane D., Attias P., Kofman T., Bouvier M., Lapidus N., Fourati S., El Karoui K., Mondor NephroCov Study Group (2021). Kinetics of anti–SARS-CoV-2 IgG antibodies in hemodialysis patients six months after infection. J. Am. Soc. Nephrol..

[37] Ben-Shachar R., Koelle K. (2015). Minimal within-host Dengue models highlight the specific roles of the immune response in primary and secondary Dengue infections. J. R. Soc. Interface.

[38] Pitt M.A., Myung I.J. (2002). When a good fit can be bad. Trends Cogn. Sci..

[39] Maucourant C., Filipovic I., Ponzetta A., Aleman S., Cornillet M., Hertwig L., Strunz B., Lentini A., Reinius B., Brownlie D. (2020). Natural killer cell immunotypes related to COVID-19 disease severity. Sci. Immunol..

